# Moving From PQRST to AI: Advancing Transparency, Reliability, and Clinical Translation in ECG Deep Learning∗

**DOI:** 10.1016/j.jacadv.2023.100682

**Published:** 2023-11-08

**Authors:** Christopher M. Haggerty, Timothy J. Poterucha

**Affiliations:** a IT Data Science, NewYork-Presbyterian Hospital, New York, New York, USA; b Department of Biomedical Informatics, Columbia University, New York, New York, USA; c Seymour, Paul, and Gloria Milstein Division of Cardiology, Department of Medicine, Columbia University Irving Medical Center and NewYork-Presbyterian Hospital, New York, New York, USA

**Keywords:** artificial intelligence, deep learning, electrocardiography (ECG), neural networks, risk prediction

**Figure:**
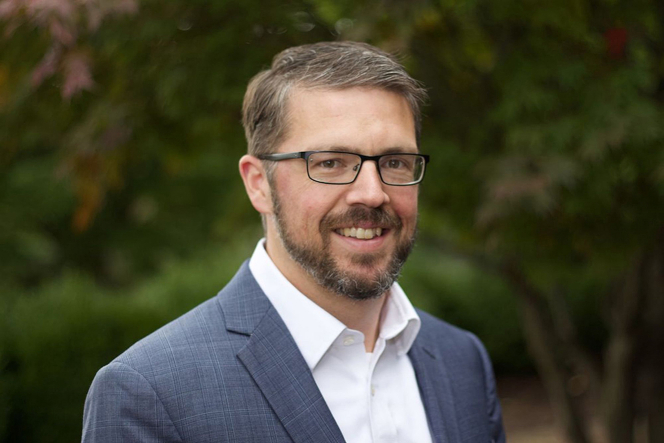

While automated evaluations of the electrocardiogram (ECG) have been around for decades, the past 5 years have seen a dramatic increase in research and development with the application of artificial intelligence (AI), particularly deep convolutional neural networks. Such models have demonstrated strong performance for a variety of tasks such as rhythm classification,^1^ detecting paroxysmal atrial fibrillation in sinus rhythm,^2^^,^^3^ detecting underlying cardiac structural or functional abnormalities^4^, ^5^, ^6^ and even risk of future mortality.^7^ These studies have generated considerable hype around the potential for ECG-AI-assisted precision medicine, taking advantage of the broad use and low cost of the ECG to help address specific diagnostic questions or perform opportunistic screening.

As this technology matures, it is important to critically assess the current state of development to help ensure that the systems produced are robust and reliable to ensure clinical impact. This is, of course, a multifaceted problem that includes considerations for clarity and transparency in reporting, replicability of findings across data sets, generalizability of performance across diverse cohorts, and careful consideration of potential domain shifts between model development and implementation.

In this issue of *JACC: Advances*, Avula et al^8^ present a systematic review of the literature on clinically-directed ECG-AI models, with a focus on standardization of methodologies, clarity in reporting, and potential for reproducibility across the field. This review identified 53 models across 44 studies through July 1, 2022. Among the findings, the authors found variability in deep learning network architecture employed (eg, use of sequential convolutional layers vs residual connection blocks vs long short-term memory units), the descriptive details presented for the included cohort(s), and the performance metrics reported. Some of the more striking findings relate to assessments of model reproducibility. For example, the evaluation of external cohort testing, which the authors broadly defined as either derived from a separate institution or from a temporally distinct period from the primary development institution, was performed for only 34% of models reviewed. Furthermore, <23% of publications reported sufficient information for model reproduction—comprising details of model architecture, convolutional layer composition, and other model hyperparameters and training data.

Based on these findings, the authors conclude that, while the performance of ECG deep-learning models has been excellent for a wide range of clinical tasks, there is a need for definition of and adherence to a standardized set of reporting guidelines. They suggest that these standards should minimally include details required for model reproduction and characteristics of the development and testing cohorts included. Notably, while some relevant standards exist,^9^^,^^10^ these standards were not designed to account for deep learning models and have important shortcomings that Avula and colleagues, as well as the authors of those tools themselves point out.^11^ Updates to these tools are reportedly forthcoming and should help address these needs.

Overall, we applaud the authors for their effort in carefully curating and evaluating details of the published models to provide these insights. Some of the findings merit more consideration and concern than others. For example, the noted variability in network architecture was interesting, but it is not clear that it represents a problem. Instead, the consistently strong performance of ECG-AI models suggests that the approach is generally robust to varied network design and hyperparameters. This variance may naturally diminish as foundational code bases, such as ‘IntroECG’,^12^ become more widely used. Future work will shed light on minimal requirements or optimal values, but that optimization will likely have only marginal impact on clinical value. On the other hand, the evidence for generally poor testing of generalizability and support for reproducibility certainly are cause for concern. Optimistically, some of these trends may be transient and already undergoing correction; that is, the backward-looking snapshot provided by this kind of review (the study inclusion period closed 15 months before publication) may not reflect the standards currently enforced on new and ongoing work in a rapidly evolving field. The included histogram showing the increasing inclusion of external testing over time is already some evidence of this trend. However, given the importance of ensuring reproducibility and generalizability of these models, continued diligence in enforcing this standard is warranted.

Finally, considering that a primary motivation and focus for this review was ensuring the reliability and clinical relevance of these models, more explicit considerations for model evaluation strategies that translate to intended clinical use are warranted. In many instances, there are inherently subtle differences between the characteristics of the patients in a model development set and the patients for whom the model is intended to be used in the “real world”. For example, detecting left ventricular dysfunction with explicit labeling requires patients who have clinically undergone both an ECG and an echocardiogram; however, the optimal patients to benefit from this model have not had an echocardiogram. These differences have important consequences for model performance as the base rates of disease will vary, often dramatically, between those 2 populations. The series of studies completed by the team at the Mayo clinic exemplified this point, as the prevalence of decreased ejection fraction (≤50%) dropped from 20.5% in retrospective model development to an observed prevalence of 1.8% in a pragmatic randomized trial.^4^^,^^13^ Anticipating such population shifts in evaluating model performance is an important and often overlooked step in translating models from retrospective development to clinical implementation. Therefore, inclusion of such considerations—even if based on assumptions with limited data—in earlier stages of model evaluation, not to mention regulatory and governance discussions, should be strongly considered to anchor performance expectations appropriately.

Where does the field go from here? First, there is currently no ability to compare the accuracy of different ECG models across studies. Standard statistical metrics, including receiver operator characteristics and precision-recall curves, fundamentally fail at this task without a shared test set. There is thus an urgent need for publication of large, deidentified ECG datasets with clinically relevant labels that will allow standardized methods of model comparison, as EchoNetDynamic and ChexNet have done for echocardiography and chest x-rays.^14^^,^^15^ Second, in-silico research can only carry us so far; more randomized control trials are needed to evaluate if these tools can have a clinical impact once they meet the heterogeneity and complexity of clinical care. Third, effective partnerships with electronic health record and information system management vendors are needed to enable effective dissemination and implementation of these ECG-based tools beyond research settings. Only once these tasks are accomplished can the impact of AI-enabled ECG analysis begin to meet the hype.

## Funding support and author disclosures

Dr Haggerty is a co-inventor of U.S. patents involving ECG deep learning. Dr Poterucha owns stock in Abbott Laboratories and Baxter International with research support provided to his institution from the Amyloidosis Foundation, 10.13039/100000968American Heart Association (Awards #23SCISA1077494 and #933452), Eidos Therapeutics, Pfidslzer, 10.13039/100006520Edwards Lifesciences, and the Glorney-Raisbeck Fellowship Award from the New York Academy of Medicine.
